# Nanocrystalline High-Entropy (Co,Mn,Ni,Cr,Fe)_3_O_4_ Spinels with Varying Fe Content for Environmental and Energy Catalysis

**DOI:** 10.3390/nano16161037

**Published:** 2026-08-20

**Authors:** Tsvetomila Lazarova, Katerina Tumbalova, Diana Kichukova, Consolato Rosmini, Grigoria Theochari, Ralitsa Velinova, Anton Naydenov, Nikolay Velinov, Genoveva Atanasova, Ivanka Spassova, Daniela Kovacheva

**Affiliations:** 1 Institute of General and Inorganic Chemistry, Bulgarian Academy of Sciences, Acad. G. Bonchev Str., Bl. 11, 1113 Sofia, Bulgaria; ktumbalova@svr.igic.bas.bg (K.T.); kichukova@svr.igic.bas.bg (D.K.); raligeorgieva@svr.igic.bas.bg (R.V.); naydenov@svr.igic.bas.bg (A.N.); genoveva@svr.igic.bas.bg (G.A.); ispasova@svr.igic.bas.bg (I.S.); didka@svr.igic.bas.bg (D.K.); 2 Institute of Organic Chemistry with Centre of Phytochemistry, Bulgarian Academy of Sciences, Acad. G. Bonchev Str., Bl. 9, 1113 Sofia, Bulgaria; consolato.rosmini@orgchm.bas.bg (C.R.); grigoria.theochari@orgchm.bas.bg (G.T.); 3 Institute of Catalysis, Bulgarian Academy of Sciences, Acad. G. Bonchev Str., Bl. 11, 1113 Sofia, Bulgaria; nikivelinov@ic.bas.bg; 4 National Centre of Excellence Mechatronics and Clean Technologies, 8 Bul. Kliment Ohridski, 1756 Sofia, Bulgaria

**Keywords:** high-entropy spinels, solution-combustion synthesis, hydrocarbon oxidation, methanol decomposition, multifunctional catalysis, nanocrystalline oxides

## Abstract

In this work, nanocrystalline high-entropy spinels with the nominal composition (Co,Mn,Ni,Cr,Fe)_3_O_4_ and different Fe contents were synthesized by a facile solution combustion method and evaluated as catalysts for the complete oxidation of light hydrocarbons and methanol decomposition. Structural characterization by XRD, SEM, TEM, XPS, Mössbauer spectroscopy, and N_2_ physisorption confirmed the formation of single-phase cubic spinels and mesoporous morphology. Increasing the Fe content resulted in changes in surface elemental distribution without altering the oxidation states of the cations. Among the investigated catalysts, HES-Fe 1:1 exhibited the highest specific surface area, favorable surface enrichment in Co and Mn, and the best catalytic performance for the complete oxidation of light hydrocarbons. In methanol decomposition, HES-Fe 1:1 showed the highest methanol conversion at lower temperatures, whereas HES-Fe 1:2 exhibited the highest CO selectivity, making it the most efficient catalyst for syngas production. The results establish a relationship between Fe content, surface composition, redox properties, and catalytic performance, revealing high-entropy spinels as promising catalysts for environmental catalysis and syngas production.

## 1. Introduction

Heterogeneous catalysis is a core of many technological processes in the production of fine chemicals, energy production, environmental protection, etc. [1,2,3]. The main catalytic agents used in these processes are expensive noble metals, which, depending on the reaction to be catalyzed, the reaction conditions, and the chemical species involved, make them easily deactivated, nonselective, and subject to limitations of various kinds. Transition-metal oxides in various combinations came to overcome these disadvantages. Transition-metal oxides are widely investigated as heterogeneous catalysts because their versatile electronic structures, arising from multiple accessible oxidation states of transition-metal cations, promote reversible redox processes, lattice oxygen activation, and oxygen vacancy formation. Nevertheless, the stability of these materials is determined by the competition between entropy stabilization and thermally driven segregation, particularly under high-temperature operating conditions [4,5].

Recently so-called high entropy oxides (HEOs) attract increased attention. HEOs are multi-element materials with a complex structure, by definition composed of at least five elements [6], enabling the creation of materials with exceptional mechanical, thermal, and catalytic properties, such as high strength, hardness, and corrosion resistance. In 2015, Rost et al. first reported the formation of single-phase solid solutions of metal oxides stabilized by their high entropy [7]. HEOs’ unique structure and chemical properties, such as thermal stability [8], magnetic properties [9], lithium-ion storage properties [10], dielectric properties [11] and catalytic properties [12], have attracted the extensive interest of researchers. Compared with the traditional doped transition-metal oxides, their chemical composition results in disordered atom distribution, affecting their structural, mechanical and chemical properties [13]. In the specific case of a spinel, the crystal structure has two different crystallographic cation sites, one with a tetrahedral and the other with an octahedral configuration. Although bivalent cations are typically considered to occupy tetrahedral sites while trivalent cations occupy octahedral sites, the presence of two cationic sub-lattices allows for the possibility of a site inversion. This property can be considered the fundamental quality on which the physicochemical properties of spinel oxides are based. In view of the above, in high-entropy spinels (HES), being multi-elemental compounds, an increase in the number of cation species would therefore affect the crystallographic site occupancy, resulting in a total or partial inversion of the two available crystallographic sites with complex distributions [14]. In fact, cation occupation in spinel oxides is largely dictated by two main factors: the crystal field stabilization energy (CFSE) determined by the oxygen ligand field and the configurational entropy (*S_config_*) [15]. According to Pan et al. [16], when the atomic percentages of a multi-element system are equal, the entire system reaches maximum configurational entropy. A similar finding was actually observed experimentally by Sarkar et al. [15] for the system with nominal formula (Co_0.2_Cr_0.2_Fe_0.2_Mn_0.2_Ni_0.2_)_3_O_4_. A compositional modulation in the variation in the configurational entropy produces changes in physicochemical characteristics and therefore makes these materials particularly interesting in heterogeneous catalysis.

For instance, Deng et al. [17] proved that the HEO (MoNiCuZnCo)_y_O_x_ not only possesses higher oxidative desulfurization efficiency but also better thermal stability compared to physically mixed multi-metal oxide catalysts. Feng et al. [18] have prepared and tested lamellar mesoporous high entropy oxides consisting of (CoNiCuMgZn)_y_O_x_ in the oxidation reaction of benzyl acid, achieving conversions of over 98% at relatively low temperatures (120 °C) and half of the reaction time compared to the classic Au/Pd-TiO_2_ catalyst. Dong et al. [19] studied high configurational entropy in (ZnNiCoMnAl)_y_O_x_ spinels. It was found that the chemical and physical characteristics of these materials appear to be particularly attractive, especially in the field of catalysis dedicated to environmental protection.

In order to get the benefits of the advantages of each component, the right synthesis technique should be chosen. Multiple methods have been employed by researchers for the synthesis of HEOs, each one having its own challenges. The most commonly reported of them are related to solid-state techniques and various wet-chemical methods [6,20,21]. Solid-state methods require prolonged high-temperature treatment and repeated grinding, often resulting in coarse particles and incomplete cation homogenization. The common wet-chemical methods, such as sol–gel and Pechini routes, involve multiple processing steps and expensive organic complexing agents, while hydrothermal synthesis relies on high-pressure autoclaves and extended reaction times. Co-precipitation is highly sensitive to precipitation conditions and may lead to compositional segregation due to differences in the precipitation behavior of individual metal ions [22,23]. The solution combustion method is a rapid, cost-effective, and energy-efficient synthetic technique for the preparation of advanced nanoscale powders. The process is based on a self-propagating exothermic redox reaction between metal salts, serving as oxidizing agents, and organic fuels, leading to the formation of highly pure and homogeneous metal oxide powders [24,25]. The modulation of configurational entropy of the spinel structure can play a significant role in determining the catalytic behavior of high-entropy spinels (HESs) under different reaction environments. While some studies reported the catalytic performance of HESs in oxidative catalytic reactions, such as alkane combustion [22,24,25,26,27], it should be noted that there is only one paper published on methanol decomposition with the use of HES materials [28].

The aim of this work is to synthesize high-entropy spinels of composition (Co,Mn,Ni,Cr,Fe)_3_O_4_ with varying Fe content using the solution combustion method, and to investigate the influence of the composition on catalytic performance in oxidative and reductive catalytic reactions, namely complete oxidation of light alkanes and methanol decomposition. The selection of Co, Mn, Ni, Fe, and Cr as constituent cations was based on their complementary structural and catalytic properties, as well as their ability to form a stable spinel lattice. All five transition metals readily adopt octahedral and/or tetrahedral coordination in spinel oxides and exhibit multiple oxidation states, which are essential for catalysis. Iron was selected as the variable component because its concentration strongly influences the cation distribution and oxidation-state equilibrium. The increase in Fe content may alter the degree of cation inversion and change the electronic interactions with other constituent cations. The novelty of the study lies in the application of a facile solution combustion synthesis route of original high-entropy spinel compositions, which enables the formation of nanocrystalline materials with promising catalytic properties for environmental and energy-related applications.

## 2. Materials and Methods

### 2.1. Materials Preparation

Finely powdered (Co,Mn,Ni,Cr,Fe)_3_O_4_ materials were synthesized via the solution combustion method. As metal precursors (oxidizers) in the synthesis reaction were used Co(NO_3_)_2_·6H_2_O (Sigma-Aldrich, St. Louis, MO, USA), Mn(NO_3_)_2_·4H_2_O (Sigma-Aldrich, St. Louis, MO, USA), Fe(NO_3_)_3_·9H_2_O (Sigma-Aldrich, St. Louis, MO, USA), Ni(NO_3_)_2_·6H_2_O (Sigma-Aldrich, St. Louis, MO, USA) and Cr(NO_3_)_3_·9H_2_O (Sigma-Aldrich, St. Louis, MO, USA). Metal nitrates were taken in molar ratios of Co:Mn:Ni:Cr:Fe as 1:1:1:1:1; 1:1:1:1:2 and 1:1:1:1:3. The samples were denoted as HES-Fe 1:1 (Co:Mn:Ni:Cr:Fe = 1:1:1:1:1); HES-Fe 1:2 (Co:Mn:Ni:Cr:Fe = 1:1:1:1:2) and HES-Fe 1:3 (Co:Mn:Ni:Cr:Fe = 1:1:1:1:3). The required amounts of metal nitrate precursors and fuel used for the preparation of high-entropy spinel oxides with different Fe ratios were as follows: Co(NO_3_)_2_·6H_2_O (0.010 mol), Mn(NO_3_)_2_·4H_2_O (0.010 mol), Ni(NO_3_)_2_·6H_2_O (0.010 mol), Cr(NO_3_)_3_·9H_2_O (0.010 mol), and Fe(NO_3_)_3_·9H_2_O (0.010 mol) for the HES-Fe1:1 composition. For the HES-Fe1:2 and HES-Fe1:3 compositions, the amount of Fe(NO_3_)_3_·9H_2_O was increased to 0.020 mol and 0.030 mol, respectively.

The configurational entropy (ΔS_mix_) of an ideal mixture of n components is calculated using the formula: ΔS_mix_ = −R∑ x_i_. ln x_i_, where R is the universal gas constant (8.314 J/mol·K) and x_i_ is the mole fraction of each component. The calculated configurational entropies are as follows: for HES-Fe 1:1—13.38 J/mol (ΔS_mix_ = 1.609R), for HES-Fe 1:2—12.98 J/mol (ΔS_mix_ = 1.561R) and for HES-Fe 1:3—12.27 J/mol (ΔS_mix_ = 1.476R), respectively. As can be seen, with increasing iron content in the composition, the configurational entropy decreases. The configurational entropy of the latter composition is slightly below the conventional threshold for high entropy oxides (ΔS_mix_ > 1.5R) proposed by Rost et al. [7]. Nevertheless, because the deviation is marginal and all compositions belong to the same compositional series, they are collectively referred to throughout this work as high-entropy spinels for the sake of simplicity.

In this synthesis, sucrose C_12_H_22_O_11_ (Valerus, Sofia, Bulgaria) (0.0125 mol for HES-Fe1:1, 0.0146 mol for HES-Fe1:2, 0.0167 mol for HES-Fe1:3) was used as a fuel (reducing agent). The amounts of reducing agent and oxidizers were selected according to the combustion principles described by Jain et al. [29], such that the total oxidizing power of the oxidizers equals the reducing power of the reducing agent. All precursors were dissolved in a total of 250 mL of deionized water under continuous stirring until a homogeneous solution was obtained. The water solution of the mixture of metal nitrates and the fuel was heated to 200 °C to slowly evaporate the solvent. After water removal, spontaneous ignition occurred between the oxidizers and the reducing agent. The reaction was accompanied by gas evolution. Powder residue containing mixed metal oxide was obtained after several minutes. The obtained powder was ground in an agate mortar until a fine powder was achieved. In the final step, the sample was thermally treated at 400 °C for 1 h in a muffle oven.

### 2.2. Material Characterization

Powder X-ray Diffraction (PXRD) patterns were collected in the range of 15–90° 2θ on a Bruker D8-Advance Diffractometer equipped with a Cu tube (λ = 1.5418 Å) and LynxEye detector. The evaluation of the diffraction patterns was performed using the EVA software package 4.0 in combination with the ICDD-PDF-2 database. Calculations of the unit cell parameters and the mean crystallite size were performed using Topas-4.2 program (Bruker, Karlsruhe, Germany). The diffraction peaks’ approximation was performed by means of the fundamental parameters approach implemented in the program [30].

The texture of the studied spinels (specific surface area, pore volume, and pore size distribution) was determined by low-temperature nitrogen adsorption at −196 °C using AUTOSORB iQ-C-MP-AG-AG (Quantachrome Instruments, Anton Paar, Boynton Beach, FL, USA). The specific surface area was calculated using the Brunauer, Emmett, and Teller (BET) equation, and the total pore volume and average pore diameter were determined according to the Gurvich rule at p/p_0_ ≈ 0.99. The pore-size distributions were determined by the Barrett–Joyner–Halenda method using the desorption branch of the isotherms.

The morphology of the samples was examined using a combination of scanning electron microscopy (SEM) and transmission electron microscopy (TEM). SEM images were obtained with a JEOL JSM-6390 (JEOL, Tokyo, Japan), while TEM analysis was performed on a JEOL 2100 microscope operating at 200 kV. The samples were placed on standard C/Cu grids for both techniques.

The Mössbauer measurements were performed with a Wissel (Wissenschaftliche Elektronik GmbH, Starnberg, Germany) electromechanical spectrometer working in a constant acceleration mode at room temperature (RT). A ^57^Co/Rh source (Activity ≈ 20 mCi) and α-Fe standard were used. The experimentally obtained spectra were fitted with WinNormos. The parameters of hyperfine interaction, such as isomer shift (δ), quadrupole splitting (∆), quadrupole shift (2ε), effective internal magnetic field (B_hf_), line widths (G_exp_), and relative weight (G) of the partial components in the spectra, were determined.

X-ray fluorescence (XRF) analysis was performed using a benchtop SpectroSCOUT instrument (SPECTRO Analytical Instruments GmbH, Kleve, Germany) equipped with a rhodium (Rh) X-ray tube and a silicon drift detector (SDD).

X-ray photoelectron spectroscopy (XPS) measurements were carried out using an AXIS Supra spectrometer (Kratos Analytical Ltd., Manchester, UK) equipped with Al K_α_ source (hν = 1486.6 eV). Before analysis, the samples were kept under ultra-high vacuum conditions overnight to minimize surface contamination. The binding energies were calibrated and determined with an accuracy of ±0.1 eV using ESCApe™ software (version 1.2.0.1325, Kratos Analytical Ltd., Manchester, UK). The surface atomic concentrations (at.%) were obtained by normalizing the integrated peak areas with the corresponding elemental sensitivity factors. Peak fitting and spectral deconvolution were performed using the same ESCApe™ software package.

### 2.3. Catalytic Tests on Complete Oxidation of Hydrocarbons

Tests on the catalytic activity of HES-Fe 1:1, HES-Fe 1:2, and HES-Fe 1:3 were performed in the complete oxidation of light alkanes (20% vol. % CH_4_ (99.95 vol.%) in N_2_ (99.999 vol. %), 8% vol. % C_2_H_6_ (99.95 vol.%) in N_2_ (99.999 vol. %), 15% vol. % C_3_H_8_ (99.95 vol.%) in N_2_ (99.999 vol. %) and 2% vol. % C_4_H_10_ (99.95 vol.%) in N_2_ (99.999 vol. %)) in a continuous-flow type of reactor with a gas hourly space velocity (GHSV) of 100,000 h^−1^ (Figure S1). The inlet concentrations of the hydrocarbons used were 0.10 vol. %, while the O_2_ (99.8 vol. %) concentration was 21.0 vol. %. All gaseous mixtures were balanced to 100 vol. % with N_2_ (99.99 vol. %). The catalyst bed volume was 0.5 cm^3^ (0.3 cm^3^ catalyst and 0.2 cm^3^ quartz-glass particles of the same size) and the reactor diameter of 6.0 mm (D_reactor_/D_particles_ ≥ 10). On-line gas-analyzers for CO/CO_2_/O_2_ (Maihak-Sick Mod. 710S, NDIR) and THC-FID analyzer for total organic content (Thermo FID-TG, SK Elektronik GmbH, Leverkusen, Germany) were used for the gas analyses.

### 2.4. Catalytic Tests on Methanol Decomposition

Methanol decomposition (MD) catalytic tests were conducted in a fixed-bed tubular reactor under continuous Ar-methanol flow (35 mL/min) (Figure S2). For this purpose, 100 mg of catalyst was used, diluting it with a 1/3 ratio of catalyst/glass-bits packing. Subsequently, the catalysts were subjected to methanol vapors decomposition (extracted by a stream of Ar (99.998 vol. %) from a saturator maintained thermostatically at 0 °C) from a temperature of 250 up to 400 °C. The reaction was kept operational for another five hours in stream at 360 °C, maintaining GHSV of 21,000 h^−1^. The produced gas phase was analyzed online by means of a HP 5890 gas chromatograph equipped with flame ionization and thermo-conductivity detectors and a PLOT-Q column (30 m × 0.53 mm). Methanol conversion throughout the catalytic process was calculated according to X(%) = ([X_i_] − [X_f_])/([X_i_]) × 100, where X are the moles of methanol and “i” and “f” are the moles of feed found at the beginning of the process and after each temperature-controlled ignition, respectively. Selectivities for each carbonaceous gas product were calculated by S_i_(%) = ([i])/(∑[C]) × 100, where “i” represents the concentration of the individual product while “C” the concentrations of all carbon-containing products.

## 3. Results and Discussion

### 3.1. XRD

**Figure 1:**
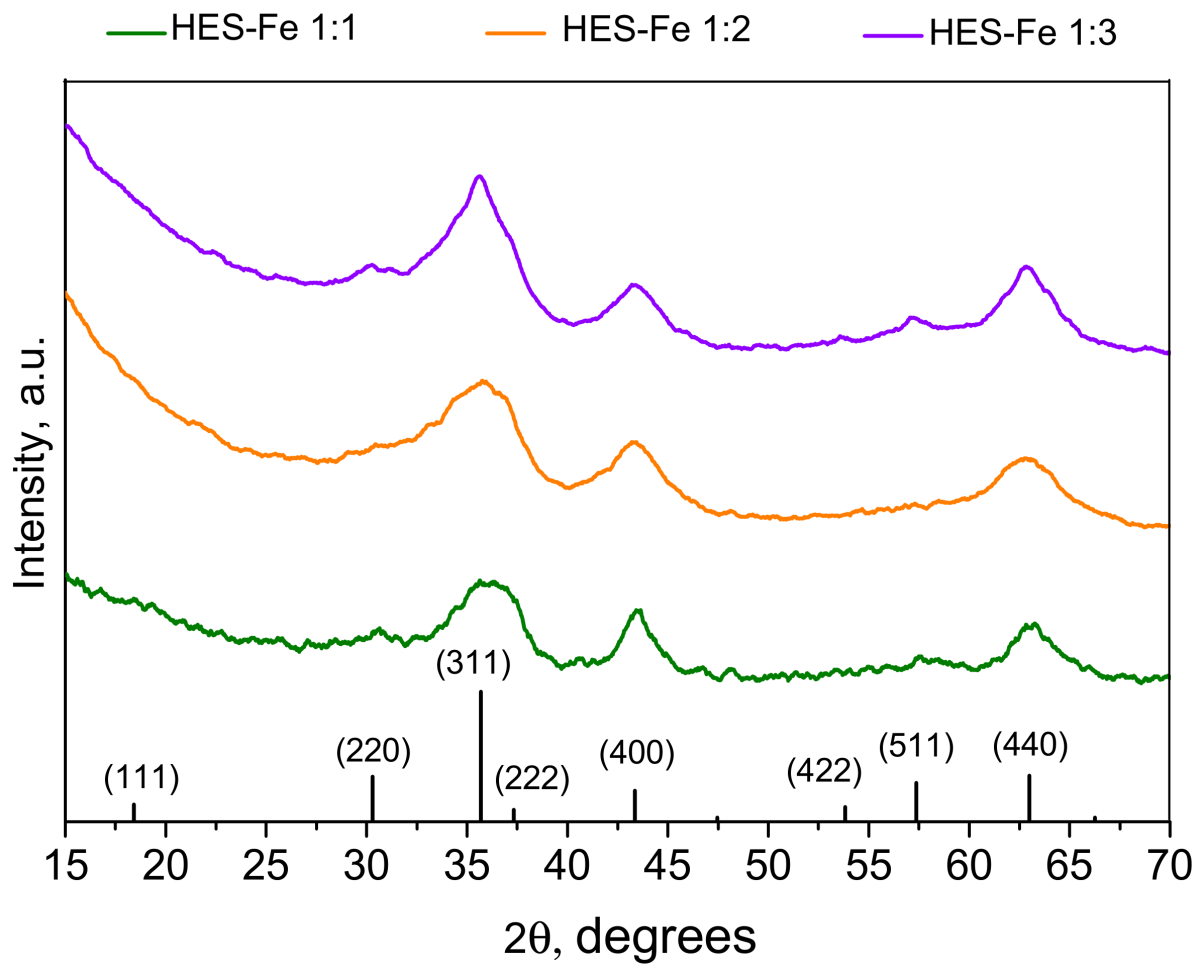
XRD patterns of the studied high entropy spinels.

The diffraction patterns of the three samples are presented in Figure 1. In all cases, the observed XRD profiles consist of broad reflections that are consistent with a cubic spinel structure with space group Fd-3m (No. 227), such as nickel ferrite (NiFe_2_O_4_, PDF 00-044-1485), whose simulated diffraction pattern is reported below in the same figure and are consistent with XRD patterns of HES reported in the literature [31]. The unit cell parameters are: for HES-Fe 1:1 a = 8.42(1)Å, for HES-Fe 1:2 a = 8.38(2)Å, for Fe-rich HES-Fe 1:3 a = 8.35(1)Å. The gradual decrease in the lattice parameter with increasing Fe content suggests a contraction of the spinel lattice, which can be attributed to a reduction in the average cationic radius caused by Fe incorporation (likely as Fe^3+^ in octahedral sites), together with possible changes in cation oxidation states of other cations and site occupancy that shorten the average metal–oxygen bond lengths. The significant peak broadening can be attributed to the effect of nanometric crystallite sizes, which are in the range of 2–3 nm.

### 3.2. Nitrogen Adsorption

**Figure 2:**
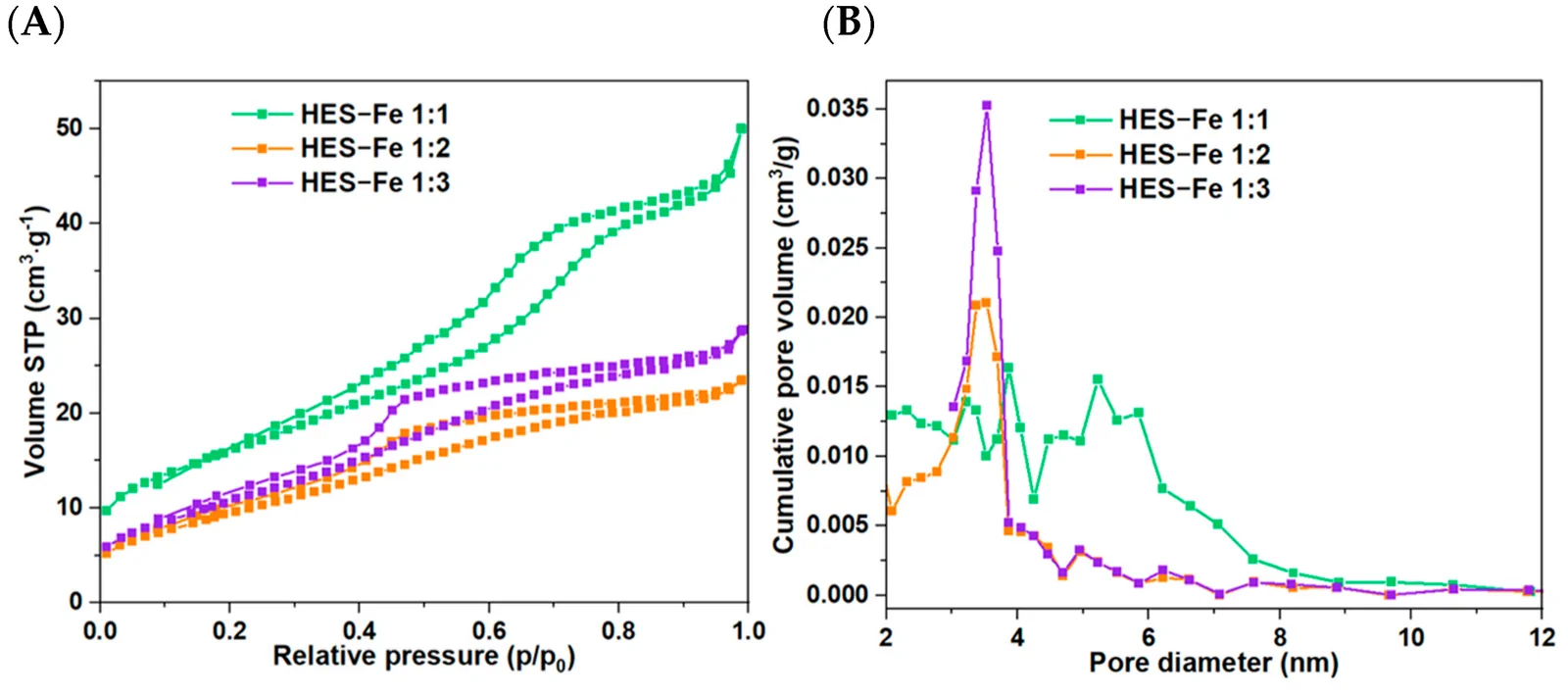
Adsorption-desorption isotherms (**A**) and pore-size distributions (**B**) of the samples under study.

N_2_ physisorption analysis was employed to evaluate the variation in textural properties of the materials as a function of cationic composition and thermal treatment applied during the synthesis procedure. Figure 2 presents the adsorption–desorption isotherms together with the pore size distribution, while the derived textural parameters are summarized in Table 1. As shown in Figure 2A, the materials exhibit type IV(a) isotherms. The HES-Fe 1:1 sample displays an H2-(b) hysteresis loop, whereas samples with higher iron content show H4-type hysteresis. According to the IUPAC report published by Thommes et al. [32], from a textural standpoint, the HES-Fe 1:2 and Fe-rich HES-Fe 1:3 catalysts can be classified as materials dominated by non-rigid mesoporosity, lacking a well-defined pore size distribution. A distinctive feature of these samples is the presence of slit-shaped pores, as indicated by nitrogen percolation at p/p_0_ < 0.4 along the desorption branch. In contrast, the HES-Fe 1:1 sample calcined at 400 °C, although still characterized by disordered mesoporosity, does not appear to exhibit significant pore entry restrictions. This observation is further supported by the pore size distribution shown in Figure 2B, particularly in the region around 3.8 nm. Likely due to this textural feature, as shown in Table 1, this sample exhibits the highest accessible surface area among the series, along with a pore volume approximately twice that of the other samples, suggesting improved pore accessibility and reduced diffusion limitations in respect its counterparts.

### 3.3. Scanning Electron Microscopy and Transmission Electron Microscopy

**Figure 3:**
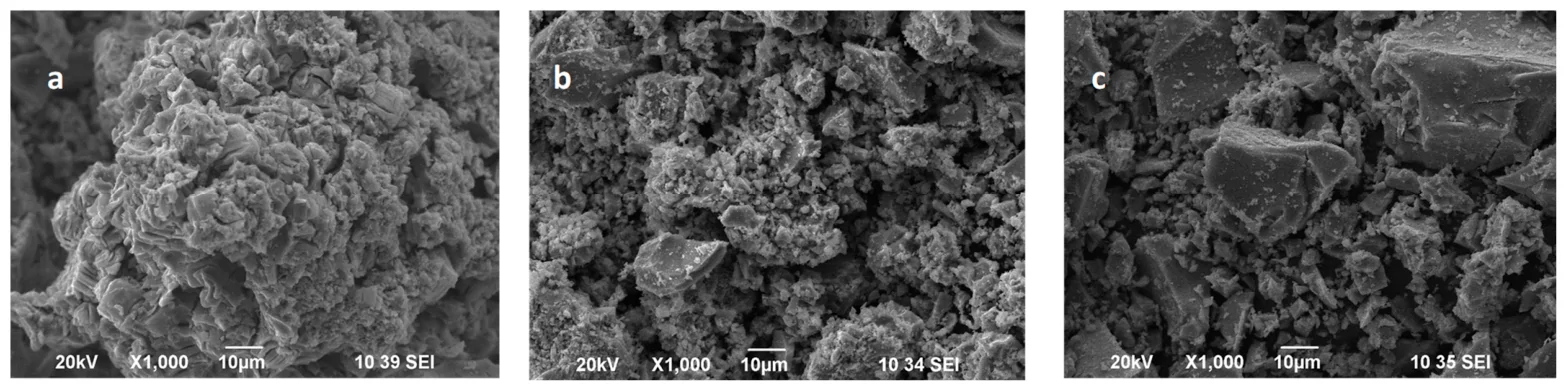
Selected SEM images of: HES-Fe 1:1 (**a**), HES-Fe 1:2 (**b**) and HES-Fe 1:3 (**c**).

The morphology of the obtained spinels is presented in Figure 3. SEM analysis reveals a clear evolution in surface morphology with increasing Fe content. All samples consist of aggregates with sizes ranging from 10 to 25 μm accompanied by a fraction with smaller size. Sample HES-Fe 1:1 exhibits a cauliflower-like agglomerated structure with a rough and porous surface, formed by the assembly of fine particles. In sample HES-Fe 1:2, the morphology becomes more irregular and fragmented, with smaller aggregates and reduced compactness. Fe-rich sample HES-Fe 1:3 shows relatively larger block-like particles with a denser and less porous appearance, indicating transition toward a more compact microstructure.

**Figure 4:**
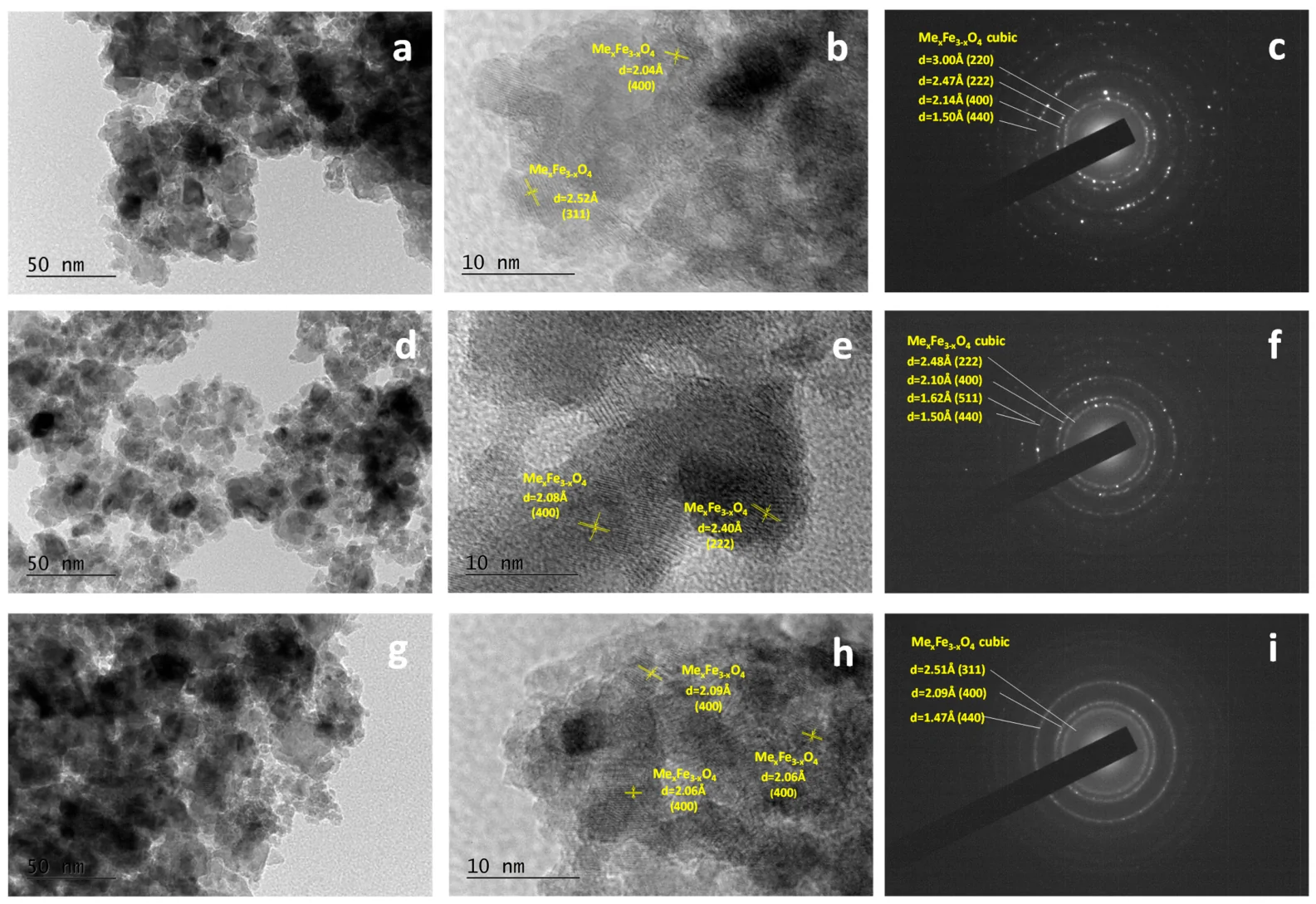
TEM micrographs at 100k magnification (**a**,**d**,**g**), HRTEM at 600k magnification (**b**,**e**,**h**), and SAED (**c**,**f**,**i**) of: HES-Fe 1:1 (**a**–**c**), HES-Fe 1:2 (**d**–**f**), HES-Fe 1:3 (**g**–**i**), respectively.

The TEM analysis of the samples (Figure 4) provides further insight into the structural differences among the three nanoparticle systems. The TEM images (Figure 4a,d,g) confirm the formation of nanoparticles with nearly spherical morphology, indicating isotropic crystal growth and nanoscale dimensions.

The particles are highly agglomerated, forming interconnected porous clusters, probably due to magnetic interactions and high surface energy. The average particle size is estimated to be within the range of 5–15 nm. Comparison with the mean crystallite size obtained from XRD suggests aggregation of a relatively small number of crystallites. The nanometric particle size and aggregated morphology provide a large surface area and abundant active sites, which may enhance the catalytic properties of these spinel ferrite materials. The HRTEM images (Figure 4b,e,h) reveal distinct lattice fringes, confirming the high crystallinity of the synthesized spinel nanoparticles. The measured interplanar distances correspond to some crystal planes, characteristic of the cubic Me_x_Fe_3−x_O_4_ spinel structure [33]. The observed d-spacing values are consistent with standard crystallographic data, verifying the successful formation of the spinel phase. The clear and continuous lattice fringes indicate good crystallinity with negligible amorphous content. No evidence of secondary phases is observed, suggesting the phase purity of the synthesized nanoparticles. The SAED patterns of the synthesized materials (Figure 4c,f,i) exhibit distinct concentric diffraction rings composed of bright spots, indicating the polycrystalline nature of the samples. These diffraction rings were indexed within the Fd-3m Space group, with d-spacings corresponding to the observed by XRD unit cell parameters, which are in good agreement with standard crystallographic data for spinel ferrites. The results are consistent with the HRTEM analysis and further verify the phase purity and crystalline nature of the synthesized materials.

### 3.4. Mössbauer Study

**Figure 5:**
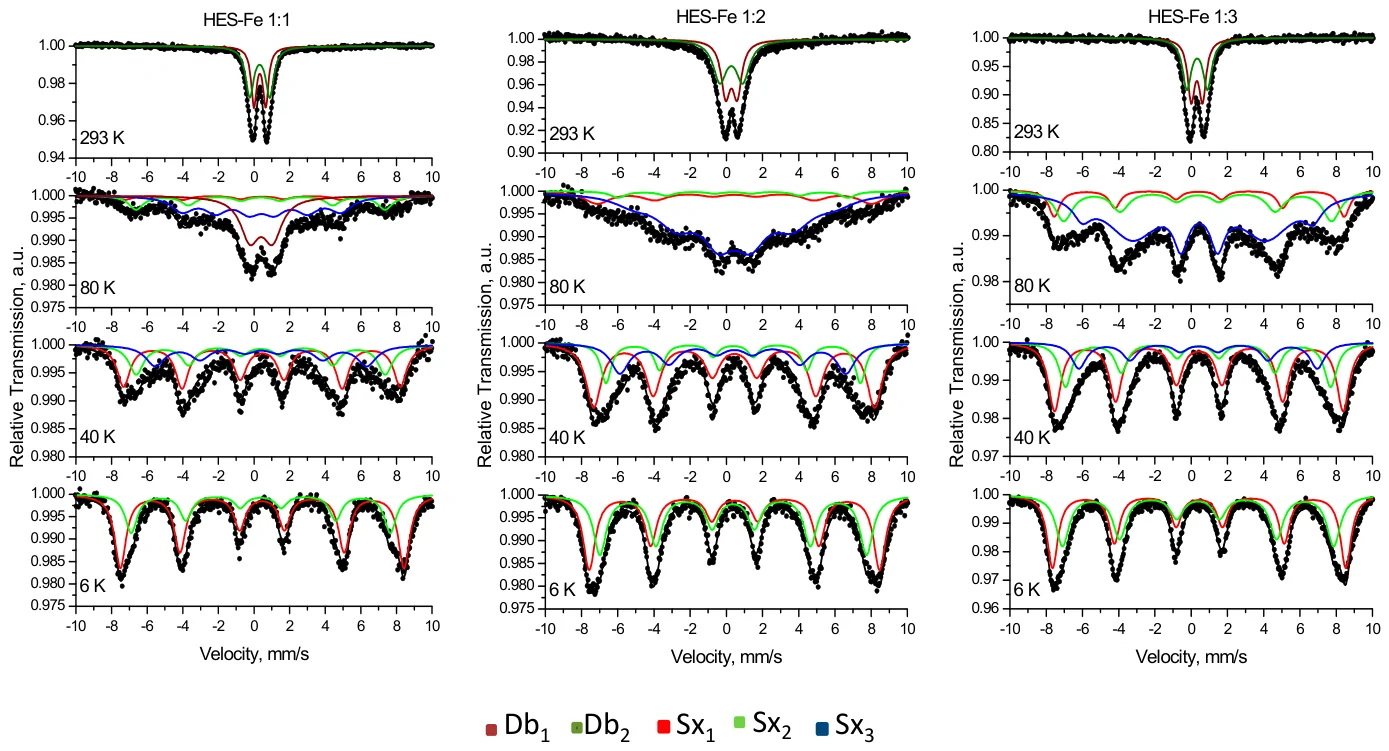
Mössbauer spectra, at 293 K, 80 K, 40 K and 6 K temperatures, of the studied HES-Fe samples.

The Mössbauer spectra of the HES-Fe samples are presented in Figure 5 and the corresponding parameters are listed in Table 2. The mathematical processing of the experimental Mössbauer spectra at 293 K was performed according to the type of spectra, using a two-doublet model. The parameters of all doublets exclusively correspond to Fe^3+^. The Db_1_ doublets, with lower quadrupole splitting values, can be attributed to iron ions in a more symmetrical environment. The Db_2_ doublets are probably due to iron in “more defective” positions, such as those located on the surface, boundaries between crystals, or iron ions in the bulk of the crystalline phase, but in a close environment of different ions. The spectra of the samples HES-Fe 1:2 and Fe-rich HES-Fe 1:3 are similar, the doublets have almost identical parameters. They differ in absorption intensity, which is due to the different iron content in the samples.

Upon cooling to 80 K, magnetic sextets emerge, reflecting the onset of magnetic ordering and the suppression of superparamagnetic relaxation. The broad distribution of hyperfine fields evidences the highly disordered cation arrangement typical of high-entropy spinels. At 40 K, the spectra are fully magnetically split and consist of three sextets corresponding to Fe^3+^ ions in tetrahedral, octahedral, and highly distorted environments. At 6 K, only two sextets remain, assigned to tetrahedral and octahedral Fe^3+^ sites, indicating complete magnetic ordering and suppression of spin fluctuations. Increasing the Fe content enhances magnetic ordering without changing the Fe^3+^ oxidation state. At the same time, the broad hyperfine field distributions across all samples confirm the preservation of the high-entropy character and significant local structural disorder [34]. A comparison between the three compositions indicates that increasing Fe concentration enhances magnetic ordering and stabilizes the magnetically blocked state. The Fe-richer samples exhibit slightly higher hyperfine fields and more balanced spectral contributions between tetrahedral and octahedral Fe^3+^ sites. This behavior suggests strengthening of Fe-O-Fe and Fe-O-M super-exchange interactions within the spinel lattice.

### 3.5. X-Ray Photoelectron Spectroscopy

**Figure 6:**
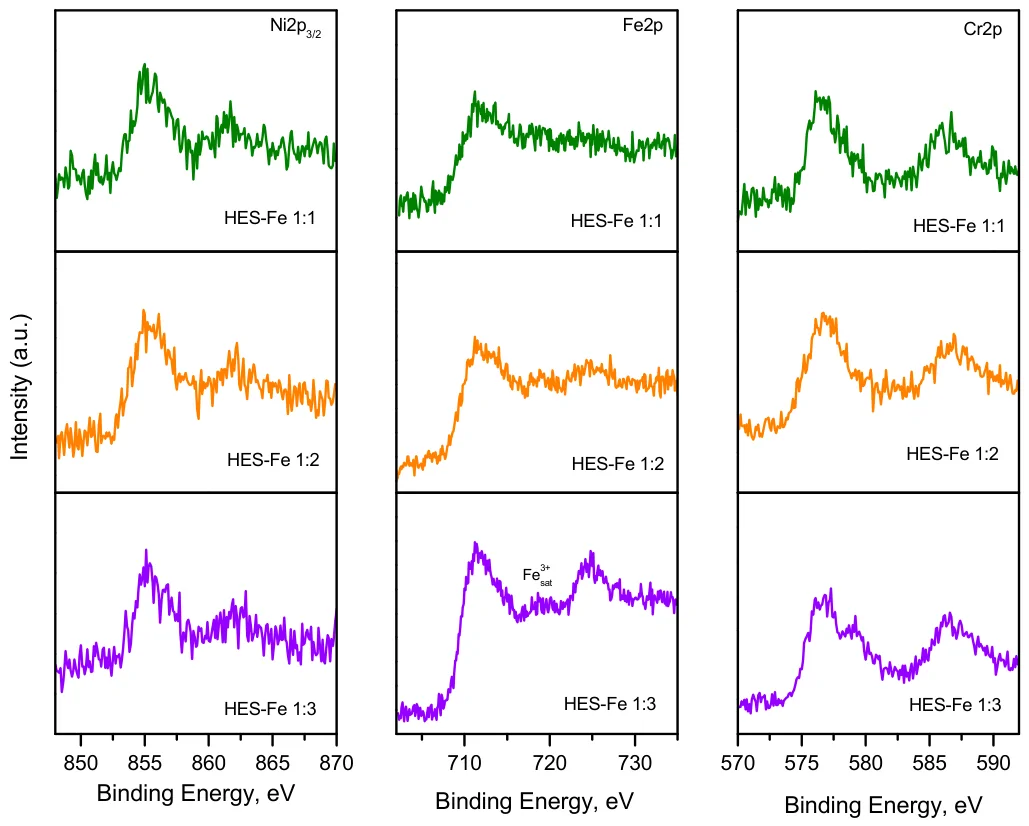
Ni 2p_3/2_, Fe 2p and Cr 2p regions of the fresh samples.

**Figure 7:**
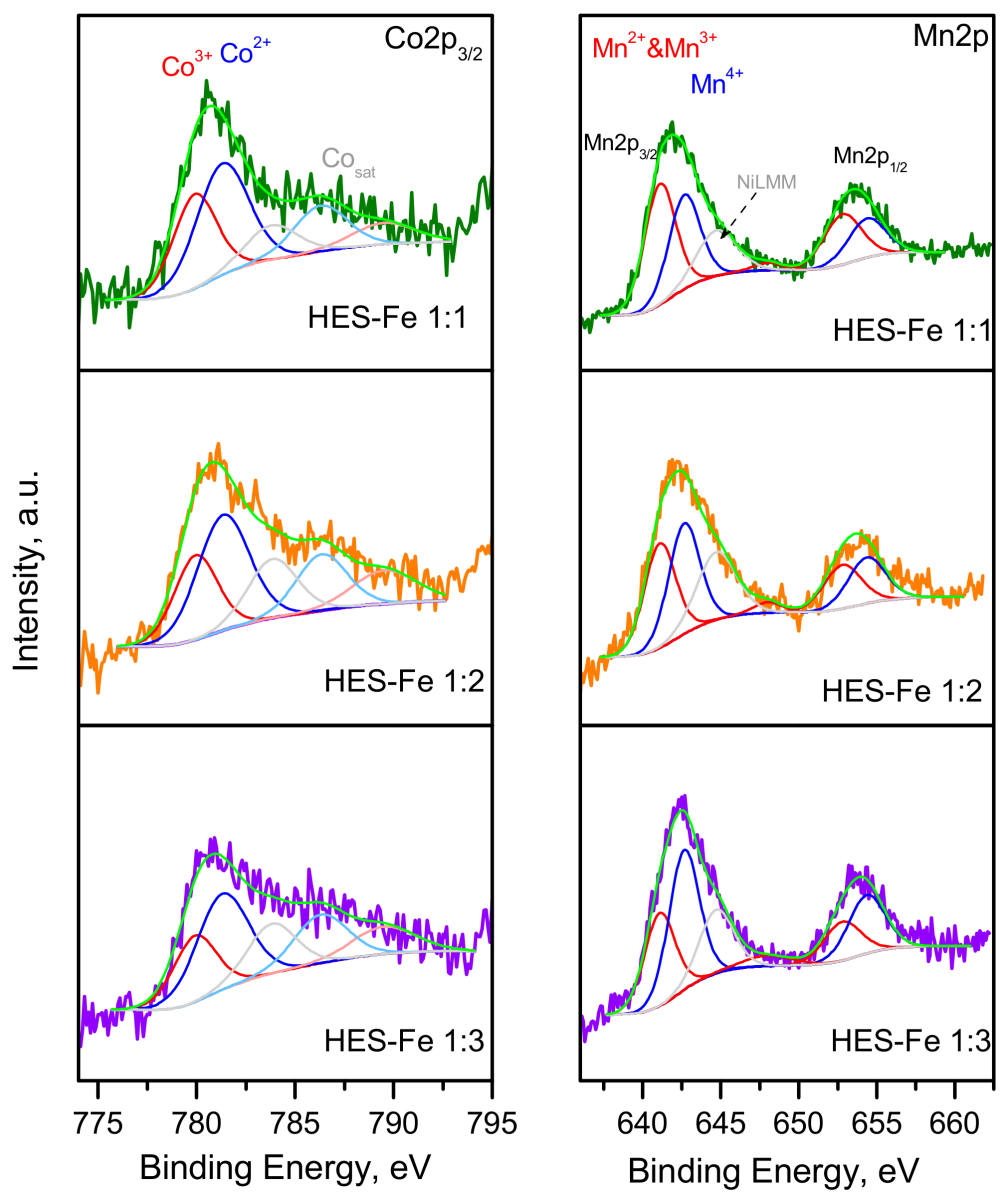
XPS spectra of the Co 2p_3/2_ and Mn 2p regions of the fresh catalysts.

The chemical states of the transition metals on the surface of the HES-Fe catalysts were investigated by XPS (Figure 6 and Figure 7). The positions of the Ni 2p_3/2_, Fe 2p and Cr 2p core levels remain nearly unchanged with increasing Fe content, indicating that the overall electronic environment of these cations is only slightly affected by the compositional variation.

The Ni 2p_3/2_ spectra for all samples exhibit a main peak centered at 855.3 eV, characteristic of Ni^2+^ species within the spinel structure. The presence of the characteristic shake-up satellite further supports the assignment of Ni in the +2 oxidation state in spinel structure [35]. No significant shift in the Ni 2p peak is observed among the three samples, suggesting that nickel maintains the same oxidation state irrespective of the iron content. The Fe 2p_3/2_ peak is located at 711.3 eV, which is characteristic of oxidized iron, predominantly Fe^3+^, in spinel oxides. The absence of a noticeable shift in the binding energy among the three samples suggests that increasing the Fe content does not significantly modify the oxidation state or the local chemical environment of iron [35,36]. This confirms the results from the Mössbauer studies. The Cr 2p_3/2_ spectrum displays a peak at 576.6 eV, corresponding to Cr^3+^, which is the stable oxidation state of chromium in normal and inverse spinel structures. The absence of components at higher binding energies suggests negligible amounts of Cr^6+^ species, especially for Fe-rich HES-Fe 1:3 sample [35].

The high-resolution Co 2p_3/2_ spectra for the HES-Fe samples were deconvoluted into two principal components located at 780.1 and 781.5 eV, assigned to Co^3+^ and Co^2+^, respectively, together with the characteristic satellite structure. The coexistence of Co^2+^ and Co^3+^ indicates the mixed oxidation states of cobalt, typical for spinel oxides. Such mixed oxidation states facilitate reversible electron transfer through the Co^2+^/Co^3+^ redox couple and are beneficial for oxidation reactions involving lattice oxygen [35]. The Mn 2p_3/2_ spectra were fitted with two major components at 641.3 and 642.6 eV, which are assigned to Mn^2+^/Mn^3+^ and Mn^4+^, respectively. The presence of Mn^2+^ is confirmed by a slight satellite contribution observed at about 647.5 eV [37]. The coexistence of Mn^3+^ and Mn^4+^ indicates a manganese environment that enhances oxygen mobility through the Mn^3+^/Mn^4+^ redox cycle.

XPS analyses reveal that the surface of all HES-Fe catalysts contains cations in multiple oxidation states, including Co^2+^/Co^3+^, Mn^2+^/Mn^3+^/Mn^4+^, Ni^2+^, Fe^3+^, and Cr^3+^. The presence of these active species provides a favorable configuration for efficient electron transfer and enhances the mobility and activation of the lattice oxygen. Nearly the same binding energies were observed for all three samples, further indicating that varying the Fe content primarily affects the relative content of the cations on the surface rather than their oxidation states.

Further insight into the chemical characteristics of the prepared spinel catalysts could be obtained by comparing the transition-metal content in the bulk and at the surface. The total elemental composition at the surface of fresh HES-Fe catalysts is shown in Table S1. The comparison of the atomic composition (normalized to 100%) of five metals (Co, Ni, Mn, Fe, and Cr) derived from XRF and XPS analyses for high-entropy spinels with different Fe content is presented in Table 3.

The elemental compositions of the HES-Fe samples determined by XRF and XPS reveal significant changes in both the bulk and surface with increasing Fe content. For the HES-Fe 1:1 sample, the XRF results show a nearly equiatomic bulk composition, indicating a relatively homogeneous elemental distribution throughout the material. In contrast, the XPS analysis demonstrates a different surface composition, with pronounced enrichment of Co, Mn and Fe, whereas Ni and especially Cr are present at lower concentrations near the surface. As expected, the Fe content increases in HES-Fe 1:2 sample, where the bulk Fe concentration rises substantially accompanied by a corresponding decrease in the concentrations of Co, Ni, Mn, and Cr. Surface analysis shows that Fe content is slightly lower than the corresponding bulk value. Co continues to exhibit significant surface enrichment, while the Mn and Ni surface concentration becomes nearly identical to its bulk concentration. In contrast, Cr remains slightly depleted at the surface compared with their bulk concentrations. Further increasing the Fe content in Fe-rich HES-Fe 1:3 sample results in the highest Fe concentrations in the bulk and at the surface, as determined by XRF and XPS, respectively. Concurrently, the concentrations of Co and Ni decrease further, consistent with their progressive substitution by Fe within the spinel structure. Unlike the lower Fe-content samples, the XPS results reveal a substantially higher Cr concentration at the surface than in the bulk, indicating pronounced surface enrichment of Cr in the Fe-rich material.

The comparison of the XRF and XPS results demonstrates clear differences between the bulk and surface elemental distributions. For the Fe-rich samples, XPS consistently reports lower Fe concentrations than XRF, suggesting that Fe is distributed throughout the bulk rather than being preferentially concentrated at the surface. In contrast, Mn exhibits consistently higher concentrations in the surface than in the bulk, indicating preferential localization of Mn to the surface. Surface enrichment of Co is observed for the HES-Fe 1:1 and HES-Fe 1:2 samples, whereas for Fe-rich HES-Fe 1:3 its surface concentration is almost equal to the bulk concentration. Ni concentration remains similar in the bulk and at the surface in all samples. The most pronounced compositional evolution is observed for Cr, which changes from strong surface depletion in HES-Fe 1:1 to clear surface enrichment in the Fe-rich HES-Fe 1:3 sample. This behavior suggests that increasing Fe incorporation promotes Cr redistribution toward the surface. Such surface compositional redistribution is typical for nanosized multicomponent spinels and is expected to influence the density and nature of catalytically active surface sites, thereby affecting their catalytic performance [38].

### 3.6. Catalytic Activity Tests on the Complete Oxidation of Hydrocarbons

**Figure 8:**
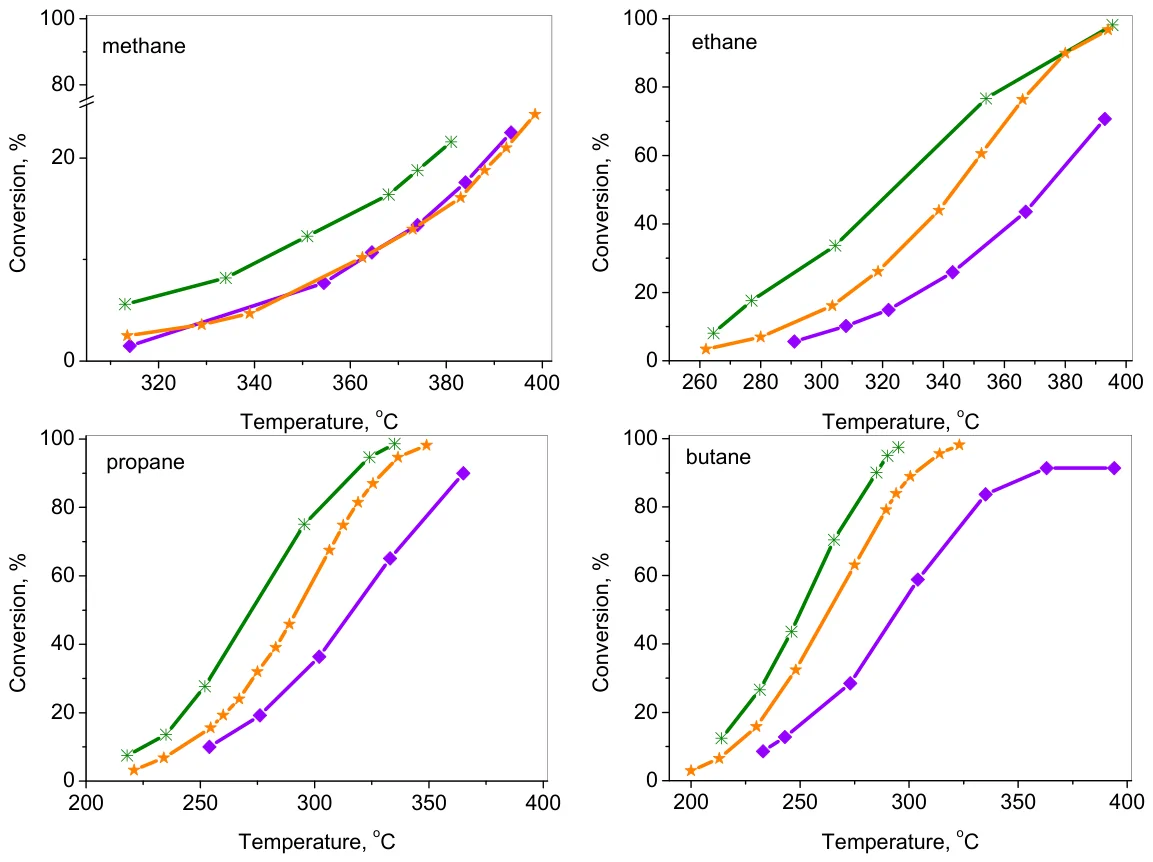
Temperature dependencies of the conversion degree during the complete oxidation of different alkanes on HES-Fe 1:1 (olive), HES-Fe 1:2 (orange), and HES-Fe 1:3 (violet).

The catalytic activity of the HES-Fe catalysts toward the complete oxidation of methane, ethane, propane, and butane was investigated as a function of temperature, and the results are presented in Figure 8. The obtained curves reveal that the catalytic behavior depends on both the hydrocarbon type and the catalyst composition. For all catalysts, the oxidation proceeds more readily with increasing alkane number, following the order methane < ethane < propane < butane.

Among the investigated samples, HES-Fe 1:1 exhibited the highest catalytic activity, with the highest conversions at lower reaction temperatures. Almost complete conversion of propane, and butane was achieved at temperatures below 350 °C. The HES-Fe 1:2 and Fe-rich HES-Fe 1:3 catalysts displayed lower activity, with their conversion curves shifted toward higher temperatures. Nevertheless, both catalysts remained highly active toward propane and butane oxidation. The methane oxidation conversion proceeds in a similar way for all HES-Fe catalysts, although the activities of HES-Fe 1:2 and Fe-rich HES-Fe 1:3 are almost equal in the detected temperature range.

“Light off” temperatures corresponding to 50% conversion (T_50_) during the complete oxidation of methane, ethane, propane, and butane over the HES-Fe catalysts with different metal ratios are presented in Table S2. For all hydrocarbons, the activity decreases with increasing Fe content, following the order HES-Fe 1:1 > HES-Fe 1:2 > HES-Fe 1:3. Methane exhibits the highest T_50_ values and is therefore the most difficult hydrocarbon to oxidize, whereas butane is the most readily oxidized, showing the lowest T_50_ values on all catalysts. The oxidation temperatures decrease in the sequence CH_4_ > C_2_H_6_ > C_3_H_8_ > C_4_H_10_, reflecting the increasing reactivity of the hydrocarbons with increasing carbon chain length [39,40]. Comparison with the recent literature (Table S3) further indicates that high-entropy spinels generally overcome conventional binary spinels [26,27,28,41,42] and some Pd-based catalysts, which are considered industrial benchmarks for VOC oxidation [43,44].

The superior VOC oxidation performance of HES-Fe 1:1 can be attributed to the synergistic interaction among the equiatomic Co, Mn, Fe, Ni, and Cr cations, which provides an optimal balance of redox-active sites and lattice oxygen mobility [45,46]. Its unique surface composition compared to Fe-richer samples exhibits pronounced surface enrichment of Co and Mn, which provide a large number of highly active Co^3+^/Co^2+^ and Mn^4+^/Mn^3+^ redox couples for oxygen activation and lattice oxygen transfer [47,48]. The nearly equiatomic bulk composition further promotes the high-entropy effect, enhancing oxygen vacancy formation and synergistic interactions among the transition metals. In addition, the relatively low surface Cr concentration avoids the formation of less-active Cr-rich surface domains in HES-Fe 1:1. The superior catalytic performance of HES-Fe 1:1 can be attributed not only to its equiatomic cation composition but also to its more favorable textural properties. As a result, HES-Fe 1:1 offers the highest density of accessible redox-active sites and the greatest oxygen mobility, leading to its superior catalytic activity for VOC oxidation. Nevertheless, all catalysts remain highly active toward light alkanes oxidation.

### 3.7. Catalytic Activity Tests on Methanol Decomposition

**Figure 9:**
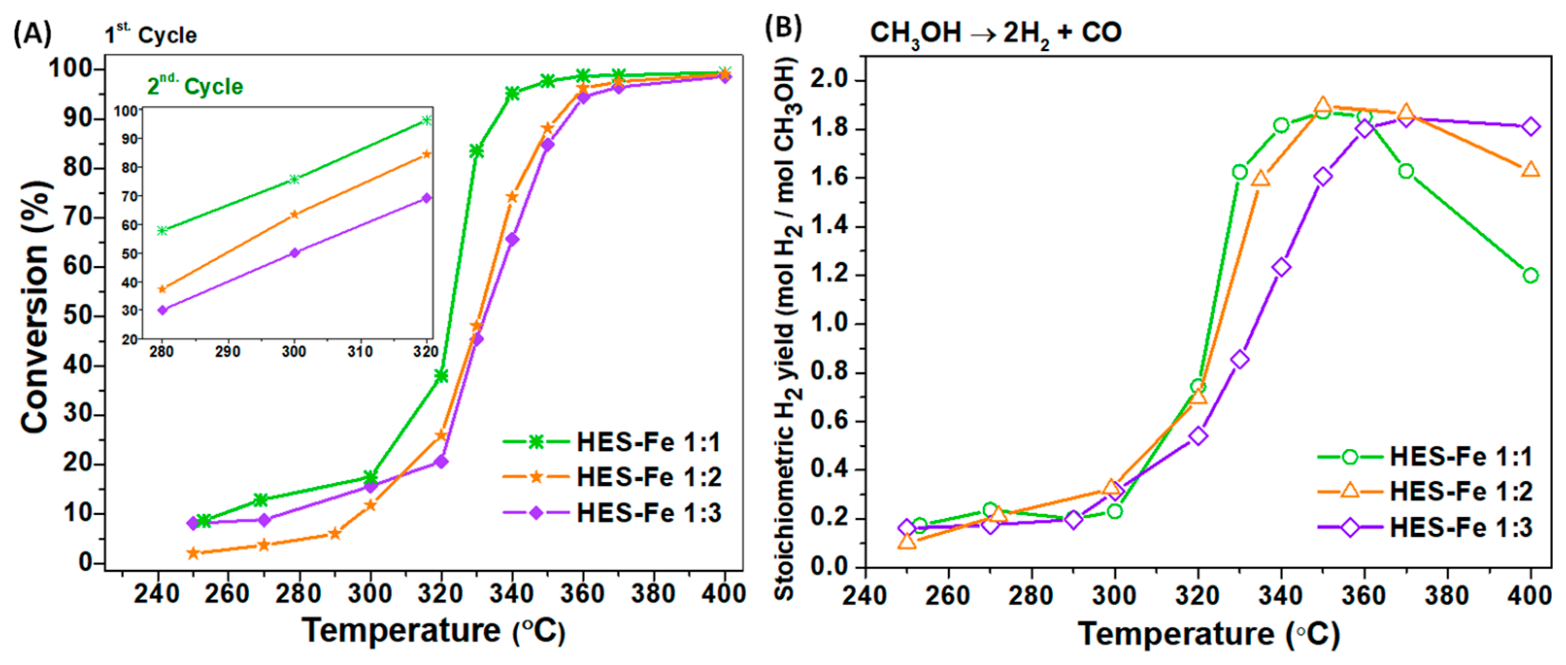
Conversion of methanol as a function of the temperature (**A**) and relative stoichiometric H_2_ yield (**B**) for the three spinels with different Fe content. *Inset:* second catalytic cycle at selected temperatures.

The three ferrous spinels with different configurational entropy were investigated in the decomposition reaction of methanol vapors to evaluate their catalytic properties, such as conversion as a function of temperature and selectivity towards the production of syn-gas. Figure 9 shows the conversion curves as a function of the operating temperature of the three spinels. It can be seen that in the first reaction cycle, the HES-Fe 1:1 spinel with the highest S_config_ converts methanol starting from lower temperatures than its analogues, reaching a conversion of 50% of methanol already at around 320 °C and 95% at 340 °C.

It is interesting to note that while the spinels HES-Fe 1:2 and Fe-rich 1:3 showed similar behavior during the first catalytic cycle, converting 50% of the methanol at around 330 °C and both 95% at around 360 °C, during the second catalytic cycle (shown in the inset of Figure 9), all catalysts showed a considerable increase in conversion and linear behavior of the same as a function of their Fe content.

The stoichiometric hydrogen yield (Figure 9B), estimated from the carbon product distribution according to the reaction stoichiometry, closely follows the methanol conversion profiles of the three catalysts. The HES-Fe 1:1 catalyst reaches the maximum H_2_ yield at lower temperatures, confirming its superior low-temperature activity associated with the highest configurational entropy. At temperatures above 350 °C, however, its H_2_ yield gradually decreases owing to the increased formation of hydrogen-consuming by-products, particularly CH_4_. In contrast, the HES-Fe 1:2 catalyst exhibits the highest maximum stoichiometric H_2_ yield (≈1.9 mol H_2_ mol^−1^ CH_3_OH), while the Fe-rich HES-Fe 1:3 catalyst displays a similar maximum value shifted toward higher reaction temperatures. Carbon mass balances were close to 100% for all catalysts over the investigated temperature range, confirming the reliability of the product quantification.

**Figure 10:**
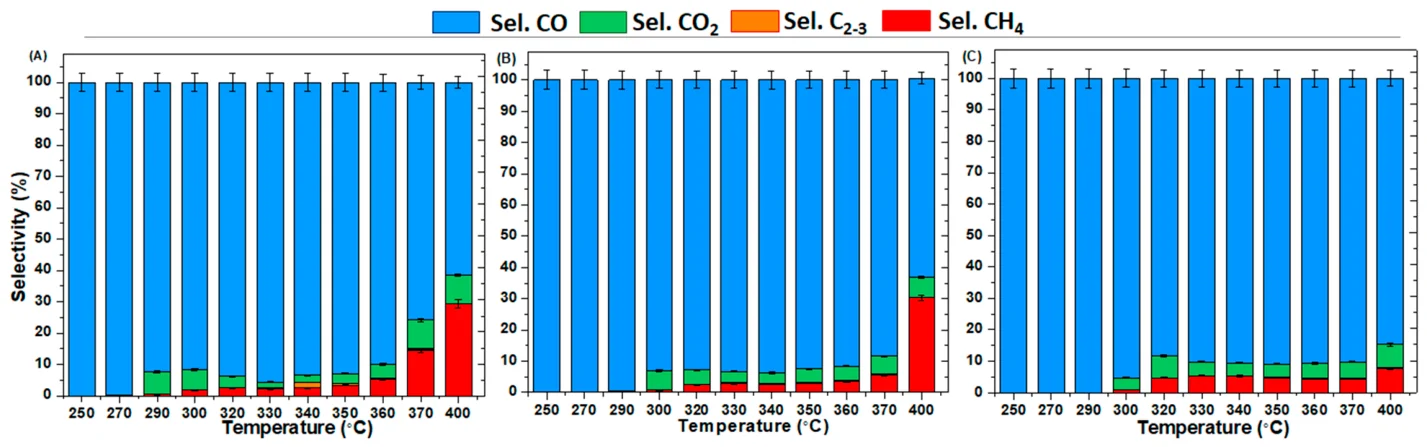
Selectivity and distribution of gas phase products as a function of operating temperature for (**A**) HES-Fe 1:1, (**B**) HES-Fe 1:2, and (**C**) HES-Fe 1:3.

Regarding the syngas selectivity, Figure 10 illustrates the evolution of the gas-phase composition for the three spinel catalysts as a function of operating temperature. All catalysts exhibit high selectivity toward CO over the entire temperature range, confirming that syngas production is the dominant reaction. At temperatures below 300 °C, CO is formed almost exclusively, while the formation of CH_4_, CO_2_, and light hydrocarbons is negligible. As the temperature increases, differences associated with the Fe content become evident. The HES-Fe 1:1 catalyst exhibits the largest decrease in CO selectivity. This decrease indicates that competing reactions occur at high temperatures. The HES-Fe 1:2 catalyst maintains the highest CO selectivity throughout the investigated temperature range, remaining close to 90% even at 400 °C. The limited formation of CO_2_ and CH_4_ suggests that this composition effectively suppresses secondary hydrogen-consuming reactions, resulting in the highest syngas selectivity among the three catalysts. Increasing the Fe content (HES-Fe 1:3) further preserves a high CO selectivity but leads to a moderate increase in CO_2_ and CH_4_ formation at elevated temperatures.

The selectivity for the methanation reaction also appears to be directly proportional to the iron cation concentration in the samples. Worthy of note is the Fe-rich HES-Fe 1:3 spinel, which even at high operating temperatures maintains selectivity for CH_4_ of no more than 8% of the gas phase composition.

**Figure 11:**
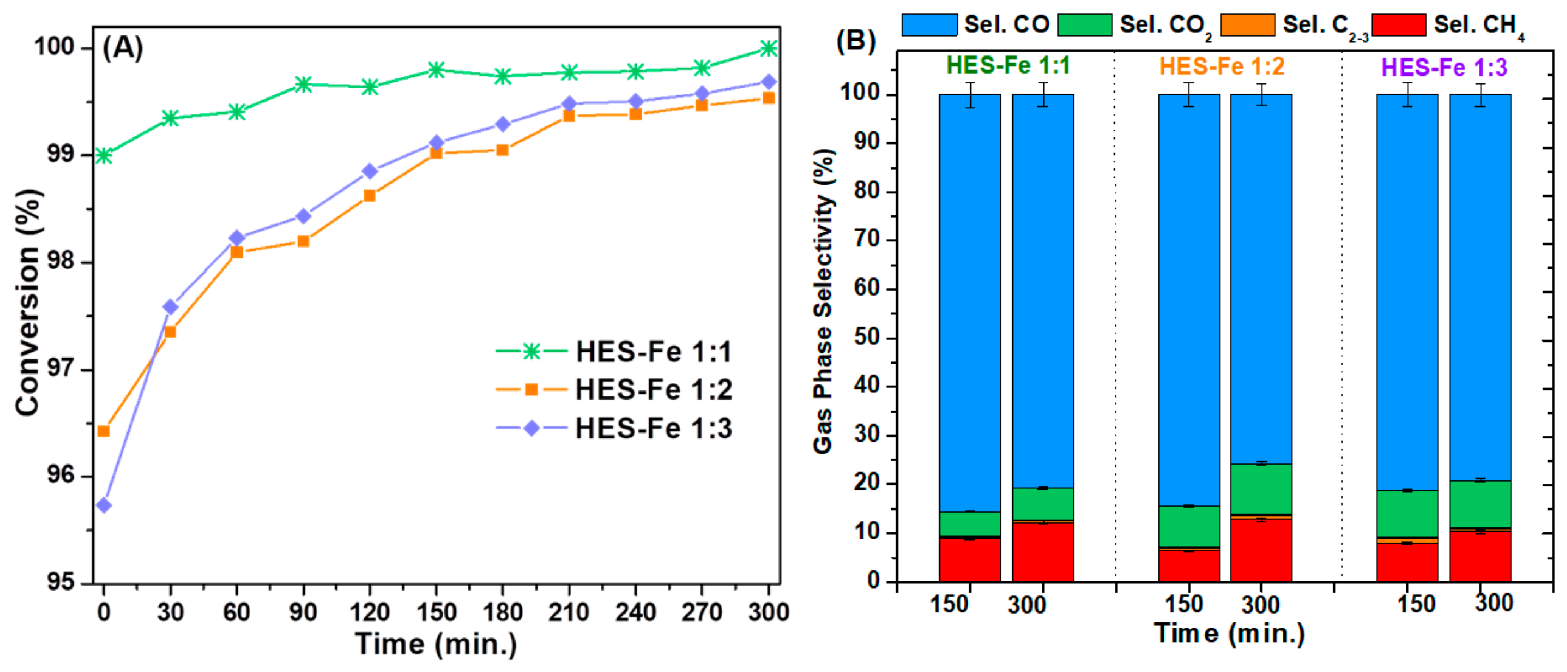
Stability test of the three high-entropy spinels at a temperature of 360 °C. (**A**) Comparison of gas phase selectivity during the stability test after 150 min of operation and after 300 min (**B**).

All samples were subjected to catalytic stability testing in the methanol decomposition reaction under conditions of total alcohol conversion, i.e., isothermal temperature of 360 °C and GHSV = 21,000 h^−1^, for a period of at least 300 min (5 h). The experimental data shown in Figure 11 confirms the phenomenon already observed in Figure 9 during the second catalytic cycle of the spinels under study. During the 5 h experiment at 360 °C, all catalysts exhibited stable performance, regardless of their initial conversion levels. Moreover, a gradual increase in conversion was observed over time, indicating catalyst activation, as reflected by the positive slope of the stability curves in Figure 11A. The gradual increase in catalytic activity observed during the stability test may be associated with progressive catalyst activation under reaction conditions. This suggests that the formation of a carbonaceous phase does not block the active sites and may possibly improve their accessibility.

### 3.8. Structural, Morphological, and Surface Changes After Catalytic Reactions

Table S1 presents the total surface composition of all fresh and spent HES-Fe 1:1 samples after methanol decomposition reaction and after VOCs oxidation. XPS analysis of the fresh HES-Fe samples revealed that carbon and oxygen are dominant elements. Following methanol exposure, the surface composition changed substantially, exhibiting a pronounced increase in carbon content and a large decrease in the detected concentrations of all metallic elements, consistent with the formation of a carbon-rich surface layer. In contrast, after VOC exposure, the surface composition of HES-Fe 1:1 remained similar to that of the fresh sample, with only moderate changes in the metal concentrations and an increase in oxygen content, suggesting a different surface interaction mechanism (Figure S3). The observed surface differences are attributed to the fundamentally different nature of the two catalytic reactions: oxidative VOC oxidation and reductive methanol decomposition.

SEM observations (Figure S4) reveal that the HES-Fe 1:1 catalyst retains its agglomerated morphology after both catalytic reactions, demonstrating high structural stability. After VOC oxidation, no appreciable morphological changes are observed, indicating negligible sintering or surface deposition under oxidative conditions. In contrast, the catalyst after methanol decomposition exhibits fine net covering portions of the particle surface. Combined with the XPS results, which show a marked increase in surface carbon, this morphology is attributed to carbonaceous deposits formed during methanol decomposition. The nitrogen physisorption of this catalyst reveals an increase in the specific surface area (61.3 m^2^/g) along with an increase in the total pore volume to 0.16 cm^3^/g and a reduction of the average pore diameter to 1 nm, probably due to the carbon contribution. Despite the presence of this carbon over-layer, the catalyst particles maintain their overall morphology, suggesting that carbon deposition is confined mainly to the surface. The XRD results support these observations by confirming preservation of the spinel structure after VOC oxidation with a small amount of MnO_2_, whereas methanol decomposition induces partial reduction of the spinel to metallic (Co,Ni,Fe) phase and carbon. These structural changes are consistent with the strongly reducing reaction environment and provide direct evidence of catalyst change during methanol decomposition.

## 4. Conclusions

Nanocrystalline high-entropy spinels with the nominal composition (Co,Mn,Ni,Cr,Fe)_3_O_4_ and different Fe contents were successfully synthesized by the solution combustion method. Structural characterization confirmed the formation of a single-phase cubic spinel structure. Mössbauer spectroscopy confirmed that iron is present exclusively as Fe^3+^, whereas low-temperature measurements revealed enhanced magnetic ordering with increasing Fe concentration. XPS analysis demonstrated that all catalysts possess a multivalent surface containing Co^2+^/Co^3+^, Mn^2+^/Mn^3+^/Mn^4+^, Ni^2+^, Fe^3+^, and Cr^3+^, providing abundant redox-active centers and promoting lattice oxygen activation. Comparison of XPS and XRF results revealed composition-dependent surface segregation, with Co and Mn enrichment on the surface of the equiatomic composition and progressive Cr enrichment at higher Fe contents, while the oxidation states of the constituent cations remained essentially unchanged.

Among the investigated materials, the HES-Fe 1:1 catalyst exhibited the highest activity for the complete oxidation of methane, ethane, propane, and butane, achieving the lowest light-off temperatures. Its best performance is attributed to the combination of the highest specific surface area, improved pore accessibility, balanced high-entropy cation distribution, and favorable surface enrichment in Co and Mn, which facilitates oxygen mobility through the Co^2+^/Co^3+^, and Mn^3+^/Mn^4+^ redox couples. Increasing the Fe content reduced the oxidation activity, mainly because of less favorable surface composition and lower textural properties. The catalysts demonstrated promising performance in methanol decomposition and stability during prolonged operation, with a gradual increase in activity, indicating catalyst activation under reaction conditions rather than deactivation by carbon deposition. HES-Fe 1:1 showed the highest methanol conversion at the lowest temperatures, whereas HES-Fe 1:2 maintained the highest CO selectivity and H_2_ yields, making it the most efficient catalyst for syngas production.

The study demonstrates a strategy for controlling the surface composition, redox behavior, and catalytic properties of high-entropy spinels. The materials combine structural stability, multivalent surface chemistry, and excellent catalytic performance, revealing their potential as efficient catalysts for VOCs abatement and syngas production.

## Figures and Tables

**Table 1:** Textural parameters acquired via N_2_-physisorption analysis.

Sample	S_BET_ (m^2^/g)	V_t_ (cm^3^/g)	D_av_ (nm)
HES-Fe 1:1	57.6	0.08	5.4
HES-Fe 1:2	36.5	0.04	4.0
HES-Fe 1:3	41.6	0.04	4.3

S_BET_—specific surface area; V_t_—total pore volume; D_av_—average pore diameter.

**Table 2:** Mössbauer parameters for the HES-Fe samples.

Sample	Temperature, K	Components	*δ*, mm/s	Δ (2*ε*), mm/s	*B_hf_*, T	*Г_exp_*, mm/s	G, %
HES-Fe 1:1	293	Db1-Fe^3+^ Db2-Fe^3+^	0.33 0.32	0.66 1.13	- -	0.37 0.54	44 56
HES-Fe 1:2	293	Db1-Fe^3+^ Db2-Fe^3+^	0.28 0.27	0.64 1.26	- -	0.60 0.90	47 53
HES-Fe 1:3	293	Db1-Fe^3+^ Db2-Fe^3+^	0.31 0.33	0.62 1.13	- -	0.47 0.58	49 51
HES-Fe 1:1	80	Db1-Fe^3+^ Sx1-Fe^3+^ Sx2-Fe^3+^ Sx3-Fe^3+^	0.40 0.45 0.37 0.43	1.27 0.00 0.00 0.00	- 47.9 43.3 27.6	1.28 1.00 1.00 1.50	33 8 18 41
HES-Fe 1:2	80	Sx1-Fe^3+^ Sx2-Fe^3+^ Sx3-Fe^3+^	0.45 0.37 0.45	0.00 0.00 0.00	47.0 39.5 34.5	1.73 1.00 2.22	17 5 78
HES-Fe 1:3	80	Sx1-Fe^3+^ Sx2-Fe^3+^ Sx3-Fe^3+^	0.43 0.36 0.40	0.03 0.00 −0.09	49.6 45.8 39.5	0.74 1.18 0.99	12 21 67
HES-Fe 1:1	40	Sx1-Fe^3+^_octa_ Sx2-Fe^3+^_tetra_ Sx3-Fe^3+^	0.45 0.38 0.40	−0.01 0.00 −0.03	48.2 43.4 36.9	0.88 1.00 1.30	48 27 25
HES-Fe 1:2	40	Sx1-Fe^3+^_octa_ Sx2-Fe^3+^_tetra_ Sx3-Fe^3+^	0.44 0.38 0.40	−0.03 0.00 −0.08	48.1 43.6 38.7	1.00 0.71 1.20	53 21 26
HES-Fe 1:3	40	Sx1-Fe^3+^_octa_ Sx2-Fe^3+^_tetra_ Sx3-Fe^3+^	0.43 0.38 0.41	−0.02 0.00 −0.06	49.4 45.4 40.1	0.83 0.83 0.99	53 28 19
HES-Fe 1:1	6	Sx1-Fe^3+^_octa_ Sx2-Fe^3+^_tetra_	0.46 0.38	0.01 0.00	49.4 45.1	0.77 0.81	67 33
HES-Fe 1:2	6	Sx1-Fe^3+^_octa_ Sx2-Fe^3+^_tetra_	0.46 0.38	−0.02 0.00	49.7 45.7	0.80 0.87	50 50
HES-Fe 1:3	6	Sx1-Fe^3+^_octa_ Sx2-Fe^3+^_tetra_	0.44 0.38	−0.01 0.00	50.3 46.2	0.75 0.83	57 43

*δ*—isomer shift; ∆ (2*ε*)—quadrupole splitting; *Г_exp_*—the line width; G—relative weight.

**Table 3:** Comparison of the atomic composition (normalized to 100%) of five metals (Co, Ni, Mn, Fe, and Cr) derived from XRF and XPS analyses for high-entropy spinels with different Fe content.

Sample	Co (At. %)		Ni (At.%)		Mn (At.%)		Fe (At.%)		Cr (At.%)	
	XRF	XPS	XRF	XPS	XRF	XPS	XRF	XPS	XRF	XPS
HES-Fe 1:1	22.0	27.8	18.9	16.4	21.4	24.7	18.6	21.7	19.1	9.4
HES-Fe 1:2	19.8	26.3	15.8	15.9	17.8	18.7	30.4	25.2	16.2	13.9
HES-Fe 1:3	15.6	15.8	13.7	10.5	15.0	17.1	46.1	29.6	9.6	17.0

## Data Availability

The data presented in this study are available upon request from the corresponding authors.

## Supplementary Materials

The following supporting information can be downloaded at https://www.mdpi.com/article/10.3390/nano16161037/s1, Figure S1: Scheme of the VOC oxidation experiment; Figure S2. Scheme of the methanol decomposition experiment; Table S1: Elemental composition on the surface of fresh and spent HES-Fe 1:1 samples; Table S2: “Light-off” temperatures (T_50_) for the synthesized HES-Fe 1:1, HES-Fe 1:2, and HES-Fe 1:3 catalysts; Table S3: Comparison of the catalytic performance of the present HES-Fe catalysts with representative high-entropy and conventional catalysts reported for methanol decomposition and light alkane oxidation; Figure S3. Survey spectra of the HES-Fe 1:1 spent samples; Figure S4. SEM images of HES-Fe 1:1 samples: (a) fresh, (b) XRD patterns of fresh and spent catalysts, (c) after VOC oxidation and (d) after methanol decomposition.
