# Supplementary material for: Nanocrystalline High-Entropy (Co,Mn,Ni,Cr,Fe)3O4 Spinels with Varying Fe Content for Environmental and Energy Catalysis
Source: Nanomaterials (Basel). 2026 Aug 20;16(16):1037. doi: 10.3390/nano16161037 (PMC13516442; doi:10.3390/nano16161037)
Supplement: Supplementary file 1 [file nanomaterials-16-01037-s001.zip › nanomaterials-4484402-supplementary.pdf]

Supplementary Materials

Nanocrystalline High-Entropy (Co,Mn,Ni,Cr,Fe)$_3$O$_4$ Spinels with Varying Fe Content for Environmental and Energy Catalysis

Tsvetomila Lazarova [1,*], Katerina Tumbalova [1], Diana Kichukova [1], Consolato Rosmini [2], Grigoria Theochari [2], Ralitsa Velinova [1], Anton Naydenov [1], Nikolay Velinov [3], Genoveva Atanasova [1], Ivanka Spassova [1,4] and Daniela Kovacheva [1,4]

[1] Institute of General and Inorganic Chemistry, Bulgarian Academy of Sciences, Acad. G. Bonchev Str., Bl. 11, 1113 Sofia, Bulgaria; ktumbalova@svr.igic.bas.bg (K.T.); kichukova@svr.igic.bas.bg (D.K.); raligeorgieva@svr.igic.bas.bg (R.V.); naydenov@svr.igic.bas.bg (A.N.); genoveva@svr.igic.bas.bg (G.A.); ispasova@svr.igic.bas.bg (I.S.); didka@svr.igic.bas.bg (D.K.)
[2] Institute of Organic Chemistry with Centre of Phytochemistry, Bulgarian Academy of Sciences, Acad. G. Bonchev Str., Bl. 9, 1113 Sofia, Bulgaria; consolato.rosmini@orgchm.bas.bg (C.R.); grigoria.theochari@orgchm.bas.bg (G.T.)
[3] Institute of Catalysis, Bulgarian Academy of Sciences, Acad. G. Bonchev Str., Bl. 11, 1113 Sofia, Bulgaria; nikivelinov@ic.bas.bg
[4] National Centre of Excellence Mechatronics and Clean Technologies, 8 Bul. Kliment Ohridski, 1756 Sofia, Bulgaria
* Correspondence: lazarova@svr.igic.bas.bg

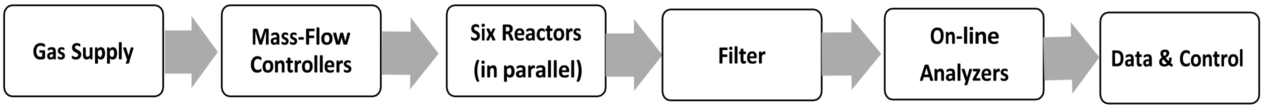

MEASURED PARAMETERS AND ANALYTICAL TECHNIQUES				
**Parameter**	**Technique/ Analyzer**	**Manufacturer/Model**	**Resolution**	**Purpose**
**VOCs** (inlet & outlet)	FID	Thermo FID, SK Elektronik, Germany	0.3 ppm VOCs (0.1 ppm as propane)	Conversion calculation
**CO_2**	NDIR	SICK, Model 710S, Maihak-Sick, Reute, Germany	0.1 ppm	Main oxidation product
**H_2O** (Water vapour)	FTIR	MKS Instruments Inc., Model 2030G, Andover, MA,USA	0.01 vol.%	Moisture in products
**CO**	NDIR	NDIR Analyzer	0.1 ppm	By-product (incomplete oxidation)
**Formaldehyde** (HCHO)	FTIR	MKS Instruments Inc., Model 2030G, Andover, MA, USA	0.1 ppm	By-product (incomplete oxidation)

Figure S1. Scheme of the VOC oxidation experiment.

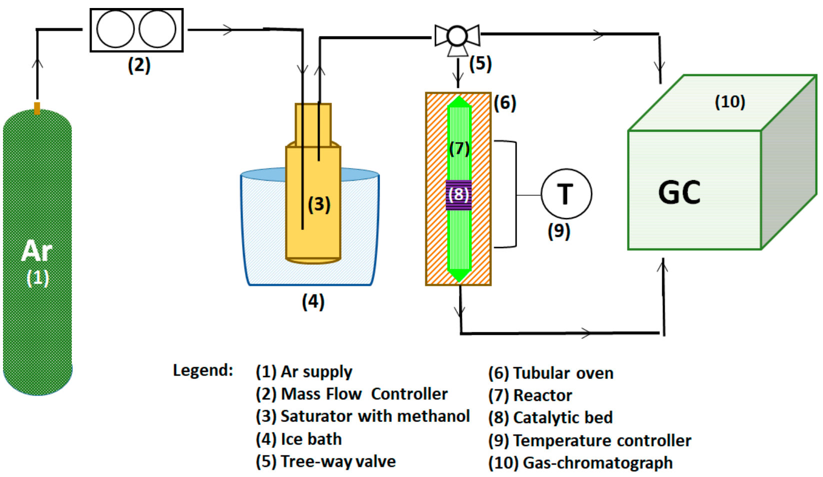

Figure S2. Scheme of the methanol decomposition experiment.

Table S1. Elemental composition on the surface of fresh and spent HES-Fe 1:1 samples.

Sample	Co [at%]	Ni [at%]	Mn [at%]	Fe[at%]	Cr[at%]	C[at%]	O[at%]
HES-Fe 1:1 fresh	7.0	4.2	6.2	5.5	2.4	40.7	34.0
HES-Fe 1:2 fresh	5.9	3.6	4.2	5.7	3.1	44.0	33.5
HES-Fe 1:3 fresh	6.3	2.6	4.2	7.2	4.2	38.0	37.5
HES-Fe 1:1 after Me-tOH	1.8	1.8	1.7	2.2	1.8	77.1	13.6
HES-Fe 1:1 after VOCs	7.3	6.2	4.3	5.0	4.8	32.0	40.4

Table S2. "Light-off" temperatures (T_{50}) for the synthesized HES-Fe 1:1, HES-Fe 1:2, and HES-Fe 1:3 catalysts.

Gas	T_{50}, ^{o}C		
	HES-Fe 1:1	HES-Fe 1:2	HES-Fe 1:3
Methane (extrapolation)	431	431	441
Ethane	323	343	373
Propane	272	292	317
Butane	251	263	295

Table S3. Comparison of the catalytic performance of the present HES-Fe catalysts with representative high-entropy and conventional catalysts reported for methanol decomposition and light alkane oxidation.

Catalyst	Catalyst type	Reaction	Catalytic performance	Stability	
HES-Fe 1:1	High-entropy spinel	Methanol decomposition	Highest CH_3OH conversion and H_2 selectivity among the synthesized catalysts at 360°C	5 h	This work
HES-Fe 1:1	High-entropy spinel	Light alkane oxidation	T_{50}(^{o}C): CH_4= 431, C_2H_6= 323, C_3H_8= 272, n-C_4H_{10}= 251	–	This work
HES-Fe 1:2	High-entropy	Light alkane	T_{50}(^{o}C): CH_4= 431, C_2H_6=	–	This

	spinel	oxidation	343, C_3H_8= 292, n-C_4H_{10}= 263		work
HES-Fe 1:3	High-entropy spinel	Light alkane oxidation	T_{50}(^oC): CH_4= 441, C_2H_6= 373, C_3H_8= 317, n-C_4H_{10} = 295	–	This work
**$(FeCrCoNiCu)_3O_4$**	**High-entropy spinel**	**Methanol decomposition**	**H_2 production rate 49.4 mmol.g^{-1}min^{-1} (solar-assisted MD, catalyst surface ≈279°C)**	**>80 h**	**29**
$(CoCrFeMnNi)_3O_4$	High-entropy spinel	Propane oxidation	Superior propane oxidation and hydrothermal stability compared with conventional spinels	Long-term	42
(Mg0.2Co0.2Ni0.2Cu0.2 $Zn_{0.2}$)Mn_2O_4	High-entropy spinel	Propane oxidation	T_{90} ≈ 260°C, excellent sulfur and water resistance	Excellent	27
Co_3O_4	Conventional spinel	Methane oxidation	Among the most active conventional spinels; low light-off temperatures attributed to highly mobile lattice oxygen	Moderate	28
$CoFe_2O_4$	Conventional spinel	Methane oxidation	Good catalytic activity but generally lower than optimized HES catalysts	Good	28
$NiCo_2O_4$	Conventional spinel	Methanol decomposition	High methanol conversion and H_2 production due to Co^{2+}/Co^{3+} and Ni^{2+}/Ni^{3+}redox couples	Moderate	28
$CoMn_2O_4$/Co-Mn spinels	Conventional spinel	Propane oxidation	Excellent propane oxidation at low temperature; activity depends strongly on Co/Mn ratio	Moderate	43
Pd/ Al_2O_3-TiO_2	Supported noble metal	Propane oxidation	proceed at temperatures above 200°C with T_{50} ≥ 320°C		44
Pd/ γ -Al_2O_3	Supported noble metal	Combustion of methane, ethane and propane	tests T_{50} = 380°C for propane combustion on 2 wt.%Pd/ γ-alumina at GHSV around 20 000 h^{-1}		45

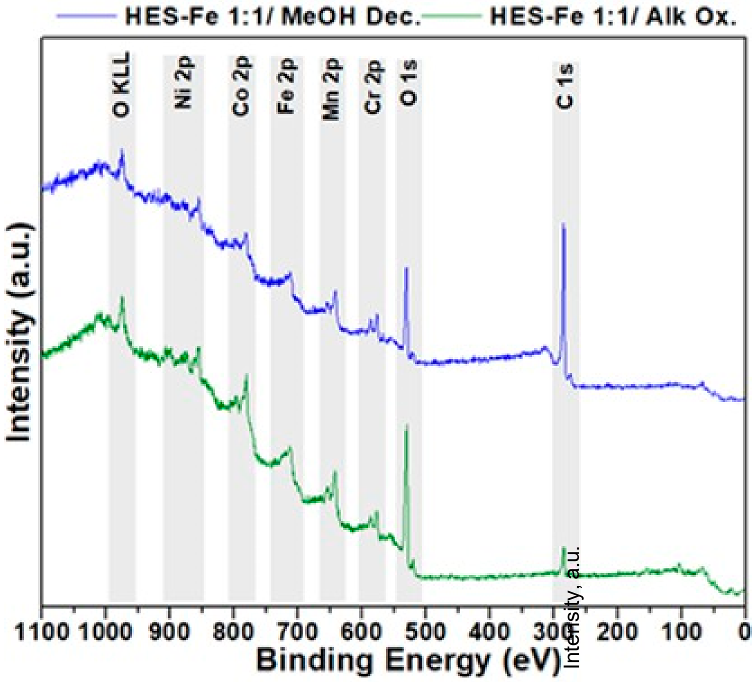

Figure S3. Survey spectra of the HES-Fe 1:1 spent samples.

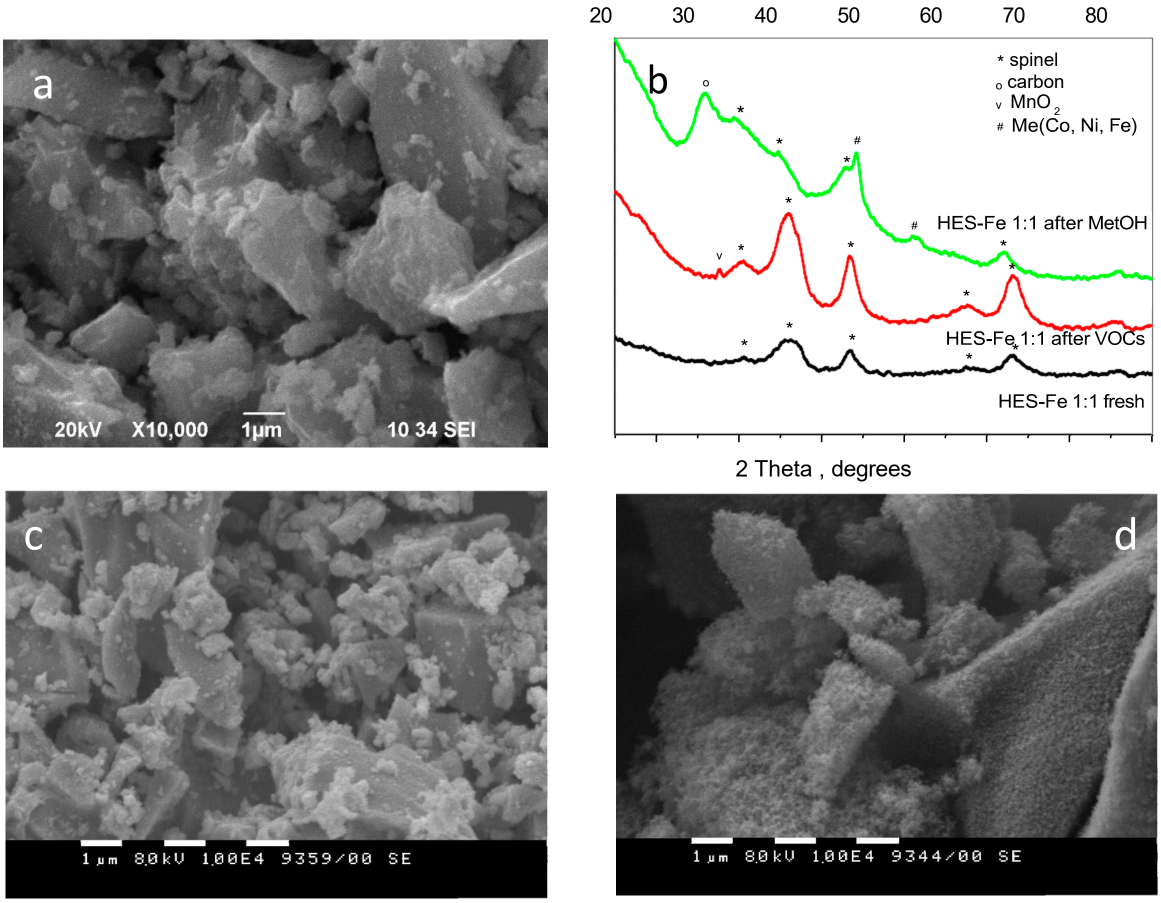

Figure S4. SEM images of HES-Fe 1:1 samples: a) fresh, b) XRD patterns of fresh and spent catalysts; c) after VOC oxidation, d) after methanol decomposition.

References:

26 Zhang, W.; Dai, W.; Zhang, W.; Yan, J.; Li, Z.; Kang, S.; Ren, S.; Lei, Z.; Wang, Z.; Shui, H. High-Entropy Mn-Based Spinel Oxides with Enhanced Catalytic Activity, Sulfur Resistance, and Thermal Stability for Propane Total Oxidation. *J. Hazard. Mater.* **2026**, *514*, 142966. https://doi.org/10.1016/j.jhazmat.2026.142966

27. Zhang, X.; Zhang, X.; Ren, L.; Han, M.; Feng, N.; Wan, H.; Guan, G. Progress and challenges of spinel materials for catalytic combustion of methane. *Fuel*, **2025**, 379, 133090. https://doi.org/10.1016/j.fuel.2024.133090

28. Tao, Y.; Wang, L.; Zhang, T.; Wang, C.; Cui, S.; Liu, B. Solar-Driven Hydrogen Production from Methanol Decomposition Catalyzed by High-Entropy Spinel Oxides. *Small* **2025**, *21*, 2500962. https://doi.org/10.1002/smll.202500962

41 Ma, Y.; Zhang, Y.; Jian, Y.; Jiang, Z.; Chai, S.; Li, L.; Xia, L.; He, C. High-entropy $(CoCrFeMnNi)_3O_4$ catalysts for propane catalytic destruction: Effect of the precipitation agent. *Fuel* **2023**, 350, 129171. https://doi.org/10.1016/j.fuel.2023.129171.

42. Zhu, W.; Chen, X.; Li, C.; Liu, Z.; Liang, C. Manipulating morphology and surface engineering of spinel cobalt oxides to attain high catalytic performance for propane oxidation. *J. Catal.* **2021**, 396, 179-191. https://doi.org/10.1016/j.jcat.2021.02.014

43. Liang, P.; Wang, L.; Wang, Q.; Zhao, B.; Li, Y.; Ma, Y.; Wei, Y.; Sun, T. Improved catalytic oxidation of propane over phosphate-modified Pd/Al_2O_3-TiO_2 catalyst. *J. Environ. Chem. Eng*. **2023**, 11, 109569. https://doi.org/10.1016/j.jece.2023.109569

44. Demoulin, O.; Le Clef, B.; Navez, M.; Ruiz, P. Combustion of methane, ethane and propane and of mixtures of methane with ethane or propane on Pd/γ-Al_2O_3 catalysts. *Appl. Catal. A Gen*. **2008**, 344, 1–9. https://doi.org/10.1016/j.apcata.2008.03.026
